# Animal Morality: What It Means and Why It Matters

**DOI:** 10.1007/s10892-018-9275-3

**Published:** 2018-09-27

**Authors:** Susana Monsó, Judith Benz-Schwarzburg, Annika Bremhorst

**Affiliations:** 1 Messerli Research Institute, University of Veterinary Medicine Vienna, Veterinärplatz 1, 1210 Vienna, Austria; 2 Division of Animal Welfare, VPHI, University of Bern, 3012 Bern, Switzerland; 3 Animal Behaviour, Cognition and Welfare Research Group, School of Life Sciences, University of Lincoln, Lincoln, LN6 7TS UK

**Keywords:** Nonhuman animals, Animal ethics, Animal morality, Moral emotions, Capabilities approach, Welfarism, Harm

## Abstract

It has been argued that some animals are moral subjects, that is, beings who are capable of behaving on the basis of moral motivations (Rowlands 2011, 2012, 2017). In this paper, we do not challenge this claim. Instead, we presuppose its plausibility in order to explore what ethical consequences follow from it. Using the capabilities approach (Nussbaum 2004, 2007), we argue that beings who are moral subjects are entitled to enjoy positive opportunities for the flourishing of their moral capabilities, and that the thwarting of these capabilities entails a harm that cannot be fully explained in terms of hedonistic welfare. We explore the implications of this idea for the assessment of current practices involving animals.

## Introduction

Rowlands (2011, 2012, 2017) has recently argued that some nonhuman animals (hereafter ‘animals’) may be moral creatures, understood as creatures who can behave on the basis of moral motivations. He has argued that, while animals probably lack the sorts of concepts and metacognitive capacities necessary to be held morally responsible for their behaviour, this only excludes them from the possibility of counting as moral *agents*. There are, however, certain moral motivations that, in his view, may be reasonably thought to fall within the reach of (at least some) animal species, namely, moral emotions such as “sympathy and compassion, kindness, tolerance, and patience, and also their negative counterparts such as anger, indignation, malice, and spite”, as well as “a sense of what is fair and what is not” (Rowlands 2012, 32). If animals do indeed behave on the basis of moral emotions, they should, he argues, be considered moral *subjects*, even if their lack of sophisticated cognitive capacities prevents us from holding them morally responsible.^1^

The empirical evidence gathered until now suggests that Rowlands may be on the right track and that some animals are indeed capable of behaving morally. Some studies, for instance, have found that animals are sometimes willing to help others when there is no direct gain involved, or even a direct loss. Such apparently altruistic behaviour has been shown by rats (Church 1959; Rice and Gainer 1962; Evans and Braud 1969; Greene 1969; Bartal et al. 2011; Sato et al. 2015), pigeons (Watanabe and Ono 1986), and several primate species (Masserman et al. 1964; Wechkin et al. 1964; Warneken and Tomasello 2006; Burkart et al. 2007; Warneken et al. 2007; Lakshminarayanan and Santos 2008; Cronin et al. 2010; Horner et al. 2011; Schmelz et al. 2017). It has further been found that some animals will offer apparent consolation to individuals in distress, a behaviour that is thought to be triggered by empathic processes and has been observed in primates (de Waal and van Roosmalen 1979; Kutsukake and Castles 2004; Cordoni et al. 2006; Fraser et al. 2008; Clay and de Waal 2013; Palagi et al. 2014), corvids (Seed et al. 2007; Fraser and Bugnyar 2010), canines (Cools et al. 2008; Palagi and Cordoni 2009; Custance and Mayer 2012), elephants (Plotnik and de Waal 2014), horses (Cozzi et al. 2010), budgerigars (Ikkatai et al. 2016), and prairie voles (Burkett et al. 2016). A few studies have also found an aversion to inequity in chimpanzees (Brosnan et al. 2005, 2010), monkeys (Brosnan and de Waal 2003; Cronin and Snowdon 2008; Massen et al. 2012), dogs (Range et al. 2008), and rats (Oberliessen et al. 2016), which suggests the presence of a sense of fairness in these species.^2^

While we believe that all this evidence provides *prima facie* support for Rowlands’ position, in this paper our aim is not to engage in an empirical or conceptual assessment of the claim that animals can be moral subjects. Rather, we shall grant that moral subjecthood in animals is at least a theoretical possibility with some empirical plausibility.^3^ Our focus, instead, is going to be on determining the ethical consequences that follow from considering that a certain animal is a moral subject.

Morality has long been understood as a feature that distinguishes humanity from the rest of the animal kingdom. It is not uncommon to find authors who use this distinguishing characteristic as a basis for denying moral rights to animals. McCloskey, for instance, argues that “[w]ithout a moral capacity, actually or potentially, there can be […] no moral exercise or waiving of a moral right, and hence no moral rights possessed by mammals that lack moral autonomy, actually and potentially” (McCloskey 1987, 79). The idea that only moral beings are entitled to moral consideration is especially salient in the contract tradition in ethics, as exemplified by the theories of Hobbes, Locke, and Kant, and can be traced back to Epicurus, who claimed: “[w]ith regard to those animals that do not have the power of making a covenant to not harm one another or be harmed, there is neither justice nor injustice” (KD, §32). This idea is also found in contemporary contractualism. For instance, Rawls states that “equal justice is owed to those who have the capacity to take part in and to act in accordance with the public understanding of the initial situation” in which the principles of justice are chosen (Rawls 1971, 505). This means that, in his view, “it is precisely the moral persons who are entitled to equal justice,” where moral persons are understood as those beings who are “capable of having […] a conception of their good,” as well as “a sense of justice” (Ibid.).

While any theory that requires individuals to be moral in order to matter morally can be questioned (see, for instance, Rowlands 2002 for a critique of the Rawlsian position), the fact remains that characterising humans as the only moral creatures may contribute to justifying a view of our species as superior to the rest, and of nature as being somehow at our disposal.^4^ This is exemplified by Machan, who states that “[n]ormal human life involves moral tasks, and that is why we are more important than other beings in nature,” a claim he uses to justify making “the best use of nature for our success in living our lives” (Machan 2002, 10–11). Any research project that explores the continuity between our species and the rest of the animal kingdom has the potential to deliver results that can serve to subvert this view of humanity, and consequently question our widespread exploitation of animals (Benz-Schwarzburg and Knight 2011; Benz-Schwarzburg 2012). Determining that morality extends beyond the human species would thus help undermine any claims of human superiority that could be used to justify the mistreatment of animals.^5^

However, we will argue that this is not the only ethical consequence attached to the idea of animals as moral subjects. It is generally assumed that the kind of ethical treatment a certain being is entitled to depends upon the *type* of being she is. While this idea has been questioned by some authors (e.g. Crary 2010), most ethicists consider that a species’ features are the cornerstone of the type of ethical treatment its members deserve. White, for example, links the very idea of ethics to the appreciation of a species’ capacities. He does so by referring to the notion of vulnerability:

Ethics—our labeling actions as ‘right’ or ‘wrong’—is grounded in the idea that the type of consciousness that we have gives us special capacities and vulnerabilities. When we label something as ‘wrong’, then, we’re saying that it crosses the line with regard to not respecting some fundamental feature that makes us human. (White 2007, 155)

It seems, indeed, plausible to consider that the ways in which members of a species can be *harmed* make them *vulnerable* in certain specific ways, and, in turn, shape the kinds of *duties* we might hold towards them. For instance, it makes no sense to say of a non-sentient being that she has a right not to be subjected to unnecessary pain. Taking this idea as our point of departure, we will argue that there is a *specific kind of harm* that can affect moral subjects *as such*, and that certain *specific rights or entitlements* follow from this. And, importantly, we will argue that this specific kind of harm cannot be captured *merely* by saying that the individual is suffering, that her experiential welfare is impaired.

In order to defend this position, we are first going to introduce what we shall term the ‘welfarist’ position in animal ethics, which we will understand in a specific, and somewhat narrow, sense, and will constitute the focus of our critique. In the next section, we will construct a hypothetical example of an animal who has the ability to behave on the basis of a certain moral motivation and, thus, qualifies as a moral subject. Using Nussbaum’s capabilities approach as our theoretical framework, we will then illustrate the kind of harm that can affect this individual *because* she possesses this motivation, and the entitlements that she has as a result of this. We will subsequently return to the welfarist position and argue that a purely welfare-oriented analysis of this individual’s case would not capture the full dimension of this harm. In the final section before concluding, we will go back into the ‘real world’ and examine some of the practices involving animals using these considerations as our guide. Our aim will be to show how humans may be interfering with the moral subjecthood of animals in a way that constitutes a violation of their entitlements.

## Welfarism and Animal Ethics

We are going to argue that the ethical implications that follow from moral subjecthood cannot be captured solely in terms of welfare. The word ‘welfare’ has many different meanings, stemming from debates in axiology, political philosophy, animal ethics, and animal welfare science. For present purposes, we will use it in a narrow sense, to signify ‘experiential welfare’ or ‘subjective quality of life,’ in the hedonistic sense of these terms. Accordingly, the position that we shall call ‘welfarism’ boils down to the idea that hedonistic welfare, or hedonistic quality of life, is the only variable that matters when it comes to measuring well-being.^6^ This means that increases in well-being are understood to correlate with an improvement in the hedonistic aspects of an individual’s life, and conversely, decreases in well-being are understood to correlate with a deterioration in these aspects. Thus, one cannot be made worse off (e.g. by having one’s freedom or autonomy taken away) unless one *feels* worse off (either immediately or as a later consequence).

Welfarism is not necessarily tied to a particular normative theory. It is a theory about what is prudentially valuable, what constitutes well-being, but it does not tell us anything about how this value ought to be pursued. Thus, there can, in principle, be both consequentialist and deontological approaches to welfarism. What characterises the form of welfarism that we are concerned with, and constitutes the focus of our critique, is the endorsement of a hedonistic account of the good, according to which the only intrinsic good is pleasure (understood, in a broad sense, to encompass both physical pleasure and psychological enjoyment), and the only intrinsic bad is pain (understood, in a broad sense, to encompass both physical pain and psychological suffering).

There are several well-known problems that follow from hedonistic accounts of the good. These problems stem from two questionable claims involved in hedonism. On the one hand, one can question that all forms of pleasure are intrinsically good, for it seems that *how* one obtains pleasure also adds to its value or disvalue. On the other hand, there appear to be many other things that we value as intrinsically good besides pleasure. This is exemplified by the classic “experience machine” thought experiment (Nozick 1974, 42–45). This machine would provide us with a non-stop flow of pleasurable experiences if we were to be plugged into it instead of living our ‘real’ lives. The fact that we would not be willing to plug ourselves into it illustrates that there are other things we value in life besides pleasurable experiences. Among the things that are also proposed as intrinsically valuable, we can find freedom, knowledge, achievement, as well as relationships of friendship, care, and love (see, e.g., Rice 2013).

Some authors have developed more sophisticated forms of welfarism that are not defeated by Nozick’s experience machine. For instance, Sumner has defended a form of welfarism that “requires that a subject’s endorsement of the conditions of her life, or her experiences of them as satisfying or fulfilling, be authentic” (Sumner 1996, 139), which means that the subject has to be autonomous and properly informed. This authenticity condition allows Sumner to escape Nozick’s objection. However, Sumner appears to not merely consider pleasure as intrinsically valuable, but also autonomy, since for him it is of high importance to preserve “the authority of welfare subjects to determine for themselves which goods they will pursue in their lives” (Ibid., 98). The value of autonomy appears to be intrinsic and not merely instrumental, for he does not simply present it as a tool to ensure enjoyable experiences, but appears to value it in itself (see, e.g. Ibid., 166 ff.). If this interpretation of Sumner is correct, then his theory does not qualify as ‘welfarist’ in the sense in which we are using the term.

Regardless of how Sumner’s welfarism is best to be interpreted, our aim here is to criticise a less sophisticated form of welfarism, in which the sole criterion for determining the well-being of an individual is the presence or otherwise of pleasure and pain, broadly construed. Welfarism, thus understood, is not very popular as an account of *human* well-being, but it is a predominant approach when evaluating animal husbandry procedures and other forms of human-animal interaction. Welfarists consider that animals are harmed by humans only in those cases in which our treatment of them generates pain or suffering, or removes opportunities for pleasant or satisfying experiences. And conversely, an improvement in the way animals are treated is thought to exist whenever there is a decrease in suffering or an increase in joyful experiences. This is especially salient in scholars that attach to the so-called ‘feelings school’ within animal welfare science, for whom “welfare is all to do with what the animal feels, with the absence of negative subjective emotional states […] and […] the presence of positive subjective emotional states” (Duncan 2004, 88).^7^ Some animal ethicists also exemplify this position, like Rollin, who has argued that “how the animal feels subjectively, what it experiences, is the key feature of welfare or well-being” (Rollin 2004, 16). A further prominent example is Ryder’s painism, which is founded on the idea that the property “that all bad things share […] is that they all cause pain (in its broad sense)” (Ryder 1999, 36), and so the aim of animal ethics “should be to reduce the pain felt by individuals” (Ryder 1999, 40).

We believe that there is some truth to welfarism. There are certain moral problems with regards to which a welfarist approach provides us with a satisfactory analysis, for it is undoubtedly the case that pleasure and pain are, respectively, good- and bad-making properties of situations, other things being equal. Moreover, there is an undeniable advantage to welfarism. For those concerned with how animals are treated, welfarism is a good position to adopt, strategically, insofar as most people will agree that subjective quality of life matters to those animals who are sentient, while the existence of other prudential values that apply to animals’ lives is a matter of some controversy. We acknowledge this advantage, but do not consider this to be sufficient reason, on its own, to fully embrace this approach. We consider that welfarism is problematic if we pretend it to account for *all* the components of a good life, and, conversely, it is also problematic if we pretend it to account for *all* the possible harms that can affect an individual. In the specific case of the moral subjecthood of animals, the ethical implications that follow from it, we shall argue, cannot be fully captured from a welfarist standpoint.

## A Thought Experiment: The Case of Sustitia

We are going to defend the claim that if one is a moral subject, then one can be subjected to a specific type of harm that (1) cannot obtain when one lacks moral subjecthood, and (2) cannot be fully explained in terms of welfare. In order to defend this idea, we will use the example of a sow that we shall call Sustitia. To facilitate our critique of welfarism, we are going to build this example in two steps. First, we will offer a characterisation of Sustitia as an individual who is being harmed in a way that can be fully captured from a welfarist perspective. We shall call her Sustitia_1_. Then, we will turn Sustitia into a *moral subject*, for the purpose of illustrating how welfarism cannot give a proper account of the ethical implications in this case. We shall call this second individual Sustitia_2_. Both Sustitia_1_ and Sustitia_2_ may resemble actual sows in certain respects, but it is very important to bear in mind that they are not meant to be *real*, or even *realistic*, sows, but rather two *hypothetical constructs* that we will use to illustrate our point.

### Sustitia_1_: A Sentient Being

Let us begin, then, with Sustitia_1_. We shall start from the assumption that Sustitia_1_ is a rather simple being, whose abilities are largely limited to the basic needs of nutrition, rest, and reproduction. What is noteworthy about Sustitia_1_ is her possession of *sentience*. This means, first of all, that she has an ability to experience physical sensations. She can experience pleasure, and she can also experience pain, where these have a subjective ‘felt’ quality to them. Pleasure *feels good* to Sustitia_1_, and pain *feels bad*. Sustitia_1_ can further experience affective states, and these too have a concrete phenomenal character. Some of these affective states are moods with no intentional object. Her happy moods and her sad moods also *feel* good and bad, respectively, to Sustitia_1_. Other affective states are emotions with intentional objects. There are things in her environment that she enjoys or feels happy *about*, and there are other things that she dislikes, that make her feel distressed, or sad, or angry, or fearful. Sustitia_1_ *experiences* different things in her environment as good or bad, depending on how they make her *feel*.

Sustitia_1_ lives on a farm. Since the moment she reached adulthood, she has been kept in a stall that is too small for her to move around freely. All she can do is stand up and lie down, which causes her stress and pain. To facilitate cleaning, the floor of her stall is slatted, which causes her claws to overgrow, resulting in painful leg and claw injuries, shoulder lesions, and teat damage. The food she is fed is low in fibre, leading to painful stomach ulcers. She is forced to urinate and defecate in the same place where she sleeps, which she finds extremely unpleasant. A couple of times per year, she is made pregnant through artificial insemination. Her human handlers are not always properly trained, and the insemination is often painful and scary. When she is about to give birth, she is put into a farrowing crate, where she will be kept for four weeks in a row, and which restricts her movements even further, causing even more distress and pain. Once the piglets are weaned, she is put back in her stall, and the cycle begins again.^8^

As we can see, Sustitia_1_ is often in pain or distressed, and this psychological and physical suffering is a direct result of the way in which her human owners keep her. Theories in applied animal ethics tend to include a prohibition against causing unnecessary pain and suffering to animals. For instance, David DeGrazia’s *principle of nonmaleficence* states that “[i]t is wrong to cause extensive unnecessary harm to others without their consent” (DeGrazia 2005; see also DeGrazia 1996, chapter 9). Given that—we are supposing—Sustitia_1_’s suffering is un-consented and extensive, as well as questionable with regard to its necessity,^9^ the practices that give rise to it should consequently be brought into question.

As things stand in this example, it seems reasonable to say that Sustitia_1_ is being *harmed* by her owners, given that these husbandry conditions lead her to suffer almost continuously. The harm that Sustitia_1_ undergoes is by no means negligible. On the contrary, a plausible case could be made to argue that a fundamental right of Sustitia_1_—the right not to be subjected to extensive and unnecessary suffering—is being violated. We do not want to lessen the importance of this harm. However, we do want to highlight that this is a harm that can be *fully* accounted for in terms that refer *solely* to Sustitia_1_’s subjective experience. Of course, one could argue that, on account of being a farm animal, Sustitia_1_ is also being harmed because she has had her freedom taken away from her, or because she is being commodified or exploited. These are fair points, but we want to bracket the harm that comes from her overall life experience and focus on the specific harm that results directly from her husbandry conditions: the inadequate flooring, the restricted space, and so on. The latter is a harm that takes the form of a subjective negative experience. Because these conditions make her suffer, and her suffering is a bad thing, Sustitia_1_ is *harmed* by them. If Sustitia_1_ were to find these living conditions pleasant or enjoyable, then they wouldn’t harm her (although, of course, it might still be wrong to exploit her). The harm that Sustitia_1_ undergoes as a direct result of her husbandry conditions *consists of* her suffering. It is a welfare problem.

### Sustitia_2_: A Moral Subject

Our characterisation of Sustitia_1_ has not provided us with any reason to think that she is a moral subject, for she has been described as a fairly simple individual with entirely self-centred interests. Now, let’s turn Sustitia into a moral subject. Accordingly, we shall now refer to her as Sustitia_2_. What makes her different from Sustitia_1_ is that Sustitia_2_ does not just suffer due to *her own* life conditions, she is also concerned with the well-being of the sows and piglets in her environment. She is surrounded by sows who are kept in the same conditions as her, and who are thus displaying continuous signs of distress. She also has to witness piglets undergoing tail-docking, teeth-clipping, and castration without anaesthesia or analgesia,^10^ and she is not indifferent to their pain and distress.

Sustitia_2_ is characterised by the possession of a mechanism in her brain that ensures that whenever she witnesses a conspecific in distress, she too undergoes a form of distress^11^ that (1) is *intentionally directed* at the distress of the conspecific, and (2) has an *urge to engage in affiliative behaviour* built into it. This means that, when Sustitia_2_ witnesses the distress of any of the conspecifics in her environment, she automatically feels distressed *about their distress*, and this reliably compels her to *comfort* them. We are going to refer to this capacity of Sustitia_2_ as her ‘sympathy’^12^ and we shall suppose, for the sake of upcoming arguments, that this is a common characteristic to her species, even though the jury is still out on this.

We can plausibly classify Sustitia_2_’s sympathy as a *moral* emotion, given that it is an emotion that has the other’s welfare as its focus (i.e., the other’s welfare is its intentional object), and motivates a response that is meant to act upon the other’s situation (i.e., Sustitia_2_ wants to improve her conspecifics’ situation, and not just her own, as was the case with Sustitia_1_). And, indeed, on account of her possession of sympathy, Sustitia_2_ now fulfils the minimal conditions put forward by Rowlands to count as a moral subject:

X is a *moral subject* if X possesses (1) a sensitivity to the good- or bad-making features of situations, where (2) this sensitivity can be normatively assessed, and (3) is grounded in the operations of a reliable mechanism (a “moral module”). (Rowlands 2012, 230)

The possession of sympathy entails a sensitivity to the morally relevant property of distress. The kind of sensitivity that Sustitia_2_ has to distress corresponds to the one Rowlands requires of moral subjects, for he establishes that “[m]oral subjects are ones who are sensitive to the good- and bad-making features of situations in the sense that they *entertain intentional content emotionally*” (Ibid., 230, emphasis by authors). Sustitia_2_’s sensitivity, in turn, can be normatively evaluated, both internally and externally. From an internal perspective, we can say that, when Sustitia_2_ comforts her conspecifics, she is doing so for morally right reasons, since her sympathy implies experiencing as bad something that is, in fact, bad (i.e., the conspecific’s distress). At the same time, from an external perspective, Sustitia_2_’s sympathy is morally good because it will tend towards alleviating her conspecifics’ distress, thus diminishing the amount of bad in the world. Moreover, her emotional reaction to others’ distress is not merely accidental or contingent. Instead, it is a systematic reaction grounded in the operations of a reliable internal mechanism—one which, we are supposing, is shared by all members of her species. Due to all this, Sustitia_2_ qualifies as a *moral subject*, in Rowlands’ sense.^13^

Now, let’s imagine that one day, Sustitia_2_ witnesses a particular piglet having his tail docked. The piglet squeals in pain, and Sustitia_2_’s sympathy kicks in. She feels distressed at the piglet’s distress, and with this feeling comes a sudden urge to engage in affiliative behaviour, in order to calm the piglet down.^14^ However, the stall that Sustitia_2_’s caretakers have placed her in acts as a physical barrier and separates her from the piglet, thus preventing her from comforting him. This situation is repeated over and over again. Whenever Sustitia_2_ sees, hears, or smells any of her conspecifics in distress, she wants to comfort them, but is prevented from doing so on account of the existence of this barrier. Given that, under normal circumstances, her sympathy would result in affiliative behaviour, we can say that the barrier prevents this moral motivation from operating *fully* or *correctly*.

As in the case of Sustitia_1_, it is undoubtedly true that Sustitia_2_’s welfare is being compromised here. Her sympathy encompasses feelings of distress, so she will suffer whenever she perceives a conspecific in distress. As before, we do not intend to lessen the importance of Sustitia_2_’s psychological suffering. However, we believe that there is *something more* going on in this example, something that can’t be specified in terms of welfare alone. We are going to argue that, in such a situation, Sustitia_2_ would be the subject of a type of harm that would not be captured by merely saying that she is suffering; that her experiential welfare is being compromised. This *something more* that is going on stems from the fact that Sustitia_2_ is being prevented from exercising her moral subjecthood. In the following section, we present a way of capturing the harm that this implies.

## Moral Emotions and the Capabilities Approach

We are going to defend the idea that any theory that focuses *solely* on welfare won’t be able to account for *all* of the problems present in the example of Sustitia_2_, and other similar ones. The defence of this idea, which will take place in Theoretical Implications: Moving Beyond Welfarism section, will rely on the use of an alternative normative framework that can capture the harm we are speaking of. Rather than attempting to build from scratch a theory that can capture our intuitions, we shall make use of a well-known theory that has already proved quite solid: the capabilities approach, which was introduced into animal ethics by Nussbaum (2004, 2007). We have chosen this theory due to (1) its individualistic character, (2) the importance it gives to social abilities, and (3) its reliance on a pluralistic theory of well-being, all of which makes it a perfect candidate to use in support of our argument.

Since a defence of this theory is beyond the scope of this paper, we will proceed by assuming its correctness. Those readers who are not entirely convinced by Nussbaum’s approach should, however, bear in mind that the use of this theory is for argumentative purposes and that alternative frameworks could also be employed here. For example, Purves and Delon (2018) have recently given an account of how animals’ lives can be meaningful that could be used to argue that a life in which an animal is allowed to exercise her moral subjecthood is more *meaningful* to her, and thus better.^15^ The integrity approach defended by Rutgers and Heeger (1999) could also be extended to argue that a life in which an animal cannot exercise her moral abilities violates the animal’s *psychological completeness*, and thus harms her. With this in mind, let us now introduce the capabilities approach, and show how it can be used to conceptualise the harm that affects Sustitia_2_ when she is prevented from exercising her sympathy.

### The Capabilities Approach

When Nussbaum introduced the capabilities approach to animal ethics, she placed much emphasis on the idea that this approach doesn’t focus merely on considerations related to pleasure and pain. Of course, this doesn’t mean that there isn’t importance attached to the idea of sentience. In fact, Nussbaum considers that the possession of sentience can be plausibly considered “a threshold condition for membership in the community of beings who have entitlements based on justice” (Nussbaum 2007, 361–362). But the capabilities approach moves beyond theories that focus solely on sentience, such as classical utilitarianism, by locating ethical significance in the existence of complex forms of life, and thus aiming to “see each thing flourish as the sort of thing it is” (Nussbaum 2004, 306).

Nussbaum considers that, for each species, there exists a series of capabilities, made up of those things that members of that species are “able to do and to be” (Nussbaum 2007, 71). Needless to say, if we are thinking of highly complex species, such as our own, the list of capabilities is immense. But out of all these capabilities, Nussbaum considers that each species has a set of “basic” capabilities, which are distinguished from the rest in that they can be “evaluated as both good and central” (Nussbaum 2004, 309), where “good” is understood as being intrinsically valuable, and “central” as being essential to the flourishing of members of that species as the sort of thing they are. In the case of human beings, the basic capabilities include some that can be shared with many other species, like “[b]eing able to move freely from place to place,” and “[b]eing able to have good health,” but also further capabilities that are species-specific, like “[b]eing able to use one’s mind in ways protected by guarantees of freedom of expression,” and “[b]eing able to form a conception of the good and to engage in critical reflection about the planning of one’s life” (Nussbaum 2007, 76–77). The key idea, for our purposes, is the following:

Because the capabilities approach finds ethical significance in the flourishing of basic (innate) capabilities—those that are evaluated as both good and central […]—it will also find harm in the thwarting or blighting of those capabilities. More complex forms of life have more and more complex capabilities to be blighted, so they can suffer more and different types of harm. Level of life is relevant not because it gives different species differential worth per se, but because the type and degree of harm a creature can suffer varies with its form of life. (Nussbaum 2004, 309)

According to the capabilities approach, then, one is harmed when the agency of another results in a *thwarting* or *blighting* of one’s basic capabilities. When a being is very complex, this doesn’t necessarily mean that she will have the capacity to suffer *more*, but it does mean that she will be capable of suffering *more types of harm* than less complex beings, given that a higher complexity means a possession of more basic capabilities that can be thwarted. And, as we shall shortly discuss, the harm that comes from the thwarting of a being’s basic capabilities doesn’t necessarily have to take the experiential form of pain or suffering.^16^

### Moral Emotions as Capabilities

Capabilities, then, are understood as those things one is able to do and to be. With this in mind, there are two ways we can characterise moral emotions in terms of capabilities, depending on whether we focus on what one is able to *do* because of them or what one is able to *be* thanks to them. If we decide to conceptualise moral emotions as primarily motivations to engage in moral behaviour, then they are not capabilities in themselves, but rather cognitive-affective mechanisms that *ground* capabilities. Thus, Sustitia_2_ might be said to have the capability *to care* for others *because* she possesses sympathy. But we can also understand moral emotions as capabilities themselves, if we were to conceptualise them as primarily character traits, that is, as dispositions to feel and behave in certain ways. Sympathy, for instance, can be understood as the capability *to be* sympathetic, that is, as a character trait that disposes one to feel distressed in the presence of others in distress and consequently engage in affiliative behaviour. Moral emotions can thus be understood as either grounding certain capabilities, or as capabilities themselves. For ease of exposition, and given that nothing turns on this largely terminological choice, we shall opt for the second conceptualisation.

Not only can moral emotions be characterised as capabilities, a subset of them—those that ground positive forms of care, such as Sustitia_2_’s sympathy—can be further characterised as *basic* capabilities. They can be conceptualised as basic capabilities because they fulfil Nussbaum’s two conditions to count as such, namely, being ‘central’ and being ‘good.’ To see this, let’s go back to our example. Because we are assuming that Sustitia_2_’s sympathy is grounded in the operations of a reliable mechanism—one that, we are supposing, is innate to her species—, we can determine that the flourishing of this capability is essential to Sustitia_2_’s functioning as the type of thing she is. At the same time, insofar as her sympathy yields an emotional and behavioural response that can be classified as the morally right one to have given the circumstances, it qualifies as a ‘positive’ or ‘good’ moral motivation. While we cannot praise Sustitia_2_ for her behaviour, given that she lacks moral responsibility, we should take into consideration that, whenever she comforts others in distress, she is doing so on the basis of a motivation that implies experiencing as bad something that *is* bad (namely, the conspecific’s distress), and so she is feeling how she *should* feel, given the circumstances. Additionally, this motivation compels her to behave in a way that is morally appropriate with respect to the situation, and generates good consequences, insofar as the conspecific’s distress will be alleviated as a result. For all this, we can say that, whenever Sustitia_2_ behaves sympathetically, “the world is—temporarily, perhaps even momentarily—a better place,” and so, that it is “a good thing that the world contains a subject like this, an individual who acts in this way” (Rowlands 2012, 254).

Not only is sympathy *instrumentally* valuable because of the good it brings to the world, it can also be plausibly considered as *intrinsically* valuable, so long as we adopt a pluralistic theory of well-being and consider one’s attachments to others, one’s relationships of love and care, as part of what it means to lead a good life. This is, in fact, the view adopted by Nussbaum herself (see, e.g., Nussbaum 2007, 345). Accordingly, those moral emotions that ground positive forms of care for others—not just sympathy, but also compassion, patience, tolerance, gratitude, etc.—are tacitly present in Nussbaum’s list of the basic capabilities of animals. Indeed, Nussbaum considers that the abilities to feel certain emotions and engage in affiliation are amongst the basic capabilities of some animals, and, correspondingly, that these animals “are entitled to lives in which it is open to them to have attachments to others, to love and care for others,” as well as “to engage in characteristic forms of bonding and interrelationship” (Ibid., 397–398). It thus seems plausible to consider Sustitia_2_’s sympathy as a basic capability of hers.

If moral emotions akin to sympathy are indeed basic capabilities, this means that the individuals who possess them are entitled to lead lives in which the exercise of these capabilities remains possible for them. We are now at a point where we can start to see the full dimension of the ethical problems implicit in the example of Sustitia_2_. As we saw, whenever an animal is treated in a way that thwarts one (or several) of her basic capabilities, she is being harmed. There are two ways in which this thwarting can occur: (1) an animal can be precluded from the possibility of exercising her capability, or (2) she can have her capability taken away from her. Sustitia_2_’s case would be an example of (1). Sustitia_2_ still possesses sympathy, but she lacks the possibility of exercising it because of the existence of a physical barrier. Despite not being able to do it, she is still *capable* of caring for others. She still has her capability, but cannot exercise it. An example of (2) might occur if Sustitia_2_ became habituated to the frequent presence of distress cues in her surroundings, to the point where she no longer felt concerned about her conspecifics. She would have become *incapable* of caring for others. She would have lost her capability. In both cases, Sustitia_2_ is being harmed by whoever has placed her in this situation, because her capability has been thwarted as a result. In the following section, we offer the reasons why the moral problems involved in the thwarting of her moral capability cannot be accounted for in terms of welfare alone.

## Theoretical Implications: Moving Beyond Welfarism

In this section, we offer four considerations that support the claim that the harm affecting Sustitia_2_ cannot be fully captured in terms of experiential welfare. Because we have not given a defence of the capabilities approach, what we will put forward cannot be considered a conclusive argument. However, its strength doesn’t solely depend on the strength of the capabilities approach, since, as we mentioned before, other theories could also be successfully employed here. The considerations we will offer are *reasons* that support the need to move beyond welfarism when analysing cases like Sustitia_2_’s. While they ultimately rely on intuitions, we hope to show that these reasons are powerful enough to cast serious doubts on the ability of welfarism to rise up to the challenge.

### Sustitia_2_ is Being Doubly Harmed

The first consideration that we can offer is that it doesn’t seem *enough* to simply say that Sustitia_2_ is suffering. In the case of Sustitia_1_, it did seem enough because we didn’t describe her as having any particular ability, over and above the ability to experience enjoyment and suffering (and to eat, sleep, and reproduce). What was wrong about the use of the farrowing crate in the case of Sustitia_1_ was not that it prevented her from building a nest for her piglets, or from comforting them if they felt distressed, because we didn’t give her these abilities in the first place. All we did was give her the ability to find it painful and stressful, which is why it’s enough to say that the farrowing crate *harms* her because it makes her *suffer*. In the case of Sustitia_2_, this treatment is also doing something else, namely, preventing her from exercising her moral capability. So simply saying that it makes her suffer isn’t enough.

Sustitia_2_, like Sustitia_1_, is harmed because her welfare is being impaired, but she is also harmed because her moral capability is being thwarted. The harm involved in the thwarting of her moral capability adds to the harm involved in her loss of welfare. Sustitia_2_ is, so to speak, doubly harmed. This doesn’t mean that the harm that affects Sustitia_2_ is on a different hierarchical order than if it were entirely specifiable in terms of experiential welfare. Our claim is, rather, that if we were to only speak in terms of welfare, we would not capture this additional harm, and so we would only have a *partial* account of how Sustitia_2_ is being wronged. But Sustitia_2_’s case is not necessarily worse than Sustitia_1_’s case. It is simply different.^17^

### Distress as a Constituent Part of Caring

A purely welfarist analysis would distract us from the fact that, under normal circumstances—i.e., if Sustitia_2_ were allowed to comfort the piglet—*her* suffering wouldn’t necessarily be a bad thing. If we accept the claim that caring for others is intrinsically valuable, then the distress that is involved in her sympathy is intrinsically valuable too, insofar as it’s part of what it means, for Sustitia_2_, to care for others. Were we to adopt a purely welfare-oriented approach to analyse this case, we could never say that suffering has intrinsic value, and so the ethical nuances of Sustitia_2_’s case would not be adequately depicted.

The capabilities approach, in contrast, allows for certain forms of suffering to be valuable in themselves, an idea that Nussbaum expresses by saying that “some animal pains may even be valuable: the grief of an animal for a dead child or parent […] may be *a constituent part of an attachment that is intrinsically good*” (Nussbaum 2007, 345; emphasis by authors). One might object to Nussbaum’s claim by arguing that an attachment can exist without grief, so grief is a *manifestation* of an attachment, not a constituent part of it. However, while it’s true that an attachment can occur without grief, it’s also true that *if* there is an attachment *and* certain conditions are met, the attachment yields grief. If it does not, then arguably there is no *real* attachment. Under certain conditions, grief is the *right* thing to feel. It’s a sign of the quality of an attachment. Whether or not this means that grief is a constituent part of the attachment seems largely a matter of stipulation, but it does seem right to say that if you value the attachment, then you have to value the grief that comes with it.

In the case of Sustitia_2_, it’s even clearer, since we haven’t given her any other social ability besides her sympathy. This is what caring *consists of* for Sustitia_2_. We cannot separate it from her distress. They are inextricably intertwined.^18^ If we want to say that her caring is intrinsically good, then we have to value the experiential form that it takes. But, of course, the fact that comforting another can constitute something valuable for a social animal has to be put into perspective, and the circumstances that give rise to this behaviour must be taken into account for a proper ethical assessment to be reached.^19^

### Harm Can Take the Form of a Habituation

One of the advantages of objective theories like the capabilities approach is that they are permeable to the fact that what one is happy or content with is largely shaped by the environment one develops in. Thus, the fact that one is happy most of the time does not necessarily mean that one’s life is going well, since one’s happiness can be the result of a process of indoctrination or manipulation, or simply a coping mechanism. This was eloquently put by Sen:

A person who has had a life of misfortune, with very little opportunities, and rather little hope, may be more easily reconciled to deprivations than others reared in more fortunate and affluent circumstances. The metric of happiness may, therefore, distort the extent of deprivation, in a specific and biased way. (Sen 1987, 45)

Likewise, in the case of animals, these coping mechanisms may develop and disguise the extent to which animals are harmed in a specific situation, which is one of the reasons why their suffering can be an unreliable measure of their well-being. If Sustitia_2_ eventually became habituated to the suffering of her conspecifics and no longer felt distressed when she perceived it, a welfarist would have to conclude that she is no longer being harmed. In fact, from a welfarist perspective, we would have to say that Sustitia_2_ has been *benefitted* due to this habituation process, insofar as it has led her to stop suffering.

Anyone who considers sympathy and caringness to be intrinsically valuable character traits must conclude that the welfarist analysis of this example is misguided. The habituation process is not beneficial, even if it results in lesser suffering. The habituation process is a further harm that is inflicted upon Sustitia_2_, because an individual who would naturally be sympathetic and caring has become callous by way of the agency of another (i.e., her human caretakers). This harm cannot be accounted for in terms of welfare. In contrast, the capabilities approach allows us to speak of harm, not only when a capability is prevented from being exercised, but also when it is taken away in its entirety. When Sustitia_2_ becomes habituated to her conspecifics’ distress, her moral capability is also being thwarted, although in a different way. We can thus continue to speak of harm, even though she is no longer suffering.

### Harm is Independent of Suffering

Welfarists were forced into a moral dilemma when a strain of blind chickens that displayed less signs of distress under crowded conditions was accidentally produced. This sparked an on-going debate on whether we should deliberately *disenhance* farm animals by use of biotechnology in order to make them incapable of suffering.^20^ The dilemma emerges because the intuitive repugnance that the biotechnological disenhancement of farm animals produces in us cannot be easily reconciled with a welfarist position. If harm depends solely on suffering, then producing animals that cannot suffer should appear as innocuous, even desirable. And yet, this seems highly counter-intuitive.

Reflecting upon the moral capabilities of animals can shed light on at least part of the reason why the biotechnological disenhancement of farm animals would be wrong. This is because the harm that comes from the thwarting of an individual’s moral capabilities occurs regardless of whether she *ever actually suffers* as a result of this treatment. To see this, consider an individual we can call Sustitia_3_. What distinguishes her from Sustitia_2_ is that, when Sustitia_3_ was an embryo, she was subjected to a process of genetic engineering aimed at depriving her of the capacity to feel distress. As a consequence of this process of disenhancement, Sustitia_3_ cannot feel distress, and consequently, she cannot feel sympathy either, for the latter is dependent on the former. From a purely welfarist perspective, Sustitia_3_ would not have been harmed by this disenhancement process, since she is, *ex hypothesi*, incapable of suffering. The capabilities approach, however, allows us to specify at least *part* of the harm inflicted upon Sustitia_3_, by saying that this disenhancement has deprived her of the capability to care for others. Sustitia_3_ has been forced into a life that contains one less type of good: a life in which she will never get to form attachments and care for others. Her life is poorer as a result, and so it is a worse life.

## Practical Implications: How Humans Interfere with the Moral Capabilities of Animals

In this section, as a last step in our argument, we will evaluate some of the human practices involving animals in light of the considerations we have made. Due to space constraints, we can just give a rough idea of the relevance of our theoretical claims for the field of applied animal ethics and human–animal interactions. Moreover, we are only going to consider those animals that are under direct human care, even though Nussbaum hints at the possibility that the capabilities approach may give rise to certain duties towards wild animals (see Nussbaum 2007, 374ff.). Throughout this section, we will often refer to certain practices that we consider ethically questionable as a whole (like the raising of animals for food), but we will assess them only with respect to the noxious effect they may have upon the moral subjecthood of the animals involved. There are many further ethical concerns with respect to these practices that are well known and have been widely discussed in the literature, but we will proceed by bracketing them and focusing on the issue at hand. This is not meant as a way of lessening the importance of these ethical concerns. Rather, our ultimate aim is to contribute a new aspect to the ethical debate surrounding these practices and perhaps strengthen the case against certain ones.

Until now, we have refrained from referring to real animals and instead used hypothetical constructs to illustrate our point. In what follows, we will refer to actual animal species whose moral capabilities have only recently begun to be studied (if at all). While we still lack the sort of evidence to confidently attribute moral capacities to them, we will proceed by assuming that they are moral subjects, in order to identify potential harms that we may be inadvertently inflicting on them. As we saw, an animal can have her moral capabilities thwarted (1) if she is precluded from the possibility of exercising them, or (2) if her moral capabilities are taken away from her. We shall now consider how these two forms of thwarting may occur in everyday human-animal interactions. For ease of exposition, we are going to divide the (1)-type cases into two groups, and consider those that are analogous to Sustitia_2_’s—because they involve animals witnessing the distress of their conspecifics and being physically prevented from intervening—separately from other (1)-type cases in which animals are deprived of further necessary pre-conditions for their moral capabilities to be exercised. We will thus refer to practices that involve humans (a) preventing animals from intervening in response to a conspecific in distress (section “Practices That Involve Animals Witnessing the Distress of Conspecifics”), (b) depriving animals from further pre-conditions for their moral capabilities to be exercised (section “Practices That Deprive Animals of Other Pre-Conditions for the Exercise of Their Moral Capabilities”), and (c) eliminating the animals’ ability to act morally (section “Practices That Eliminate the Moral Capabilities of Animals”).

### Practices That Involve Animals Witnessing the Distress of Conspecifics

Even though Sustitia_2_ was an imaginary example, her life conditions may not differ much from those of real animals who are raised for food. As we have already pointed out, painful and distressing procedures in farm animal husbandry are abundant, and indeed, ethical concerns with respect to the methods involved in breeding, raising, handling, transporting, and slaughtering farm animals have been raised for decades (e.g. Rollin 2003). Due to the overcrowding that characterises intensive farming, these painful procedures will often take place while in the presence of conspecifics. This, however, is an issue that has received comparatively little attention. Only rather recently has it begun to be systematically addressed as a research topic. For instance, it is currently debated whether pigs and other animals brought to slaughter suffer from witnessing their conspecifics’ pain and fear (Anil et al. 1996, 1997; Düpjan et al. 2011; Edgar et al. 2012; Reimert et al. 2013). As usual, the focus of these studies has been the welfare problems involved in these situations. The possibility remains, however, that these animals may be moral subjects, and that, upon witnessing their conspecifics’ distress, they experience an urge to engage in caring behaviour that they cannot fulfil due to the presence of physical barriers. As we have already explained, this might add a new dimension to the ways in which these animals are being harmed.

Farm animals are not the only class of animals under human care that are often exposed to the distress of conspecifics. Lab animals, too, will frequently find themselves in similar situations. The procedures involved in experimental set-ups include handling the subjects, collecting blood samples, performing orogastric gavage (a technique used to administer nutrients directly to the stomach via an oral tube) (Balcombe et al. 2004), restraining their movements, performing tail-vein injections, and euthanising them (Sharp et al. 2003; Boivin et al. 2016), all of which frequently cause pain or distress to the subjects. Other animals in the laboratory may have perceptual access to these processes and will most likely be prevented from interfering. We already have a significant amount of evidence suggesting that rodents undergo emotional contagion when in the presence of a conspecific in distress (Knapska et al. 2006; Langford et al. 2006; Jeon et al. 2010; Atsak et al. 2011; Burkett et al. 2016), and that, when given the choice, they will help or engage in affiliative behaviour directed at a distressed individual (Church 1959; Rice and Gainer 1962; Evans and Braud 1969; Greene 1969; Langford et al. 2010; Bartal et al. 2011, 2014; Burkett et al. 2016). Therefore, in this context it is also important to consider whether the animals are having their moral capabilities thwarted.

### Practices That Deprive Animals of Other Pre-Conditions for the Exercise of Their Moral Capabilities

The exercise of an animal’s moral capabilities will most likely depend on the existence of a stable social environment where relationships with conspecifics can take place, develop, and be maintained. The existence of such a stable social environment can thus be plausibly considered as an important pre-condition for the flourishing of an animal’s moral capabilities. Many husbandry systems resort to the re-grouping, separation, or even isolation of animals, thus depriving them of this pre-condition, and potentially thwarting their moral capabilities.

This sort of unstable social environment is very common in farms. Farm animals are grouped and re-grouped according to productivity and reproductive state. This could constitute a problem, for instance, for dairy cows, who are gregarious animals and develop complex social relationships, characterised by feeding and resting together, or by engaging in allogrooming. Gutmann et al. (2015) showed that long-term familiarity had a stronger effect on the intensity of social relationships, measured in terms of time and energy investment, than having a very recent shared experience. They conclude that it is actually long-term familiarity that creates preferred social partners in dairy cows. But if farm animals are frequently re-grouped, the only social relationships possible, then, might be short-term relationships. They lose their preferred social partners, and this may hinder the flourishing of their moral capabilities, for evidence suggests that animals have a higher probability of engaging in caring and helping behaviour when they are familiar with the other subject (Cronin 2012; Bartal et al. 2014).

Routine re-grouping is not the only procedure that causes an inadequate social environment for the flourishing of farm animals’ moral capabilities. Several of the housing conditions found in factory farms, such as sow stalls and farrowing crates, have been severely criticised, amongst other things, because they result in an enforced isolation from conspecifics (see e.g. Rollin 2011, especially chapter 15). The thwarting of the moral capabilities of these isolated animals adds a new dimension to the welfare problems that such housing methods cause.

Other animals under human care are also deprived of the stable social environment that would be a pre-condition for exercising their moral capabilities. Zoo animals are often separated from each other due to space constraints (if families become too big), and rehoused to other zoos because of breeding programs. Lab animals might be kept in sterile, single housing due to the requirements of a controlled experimental setting. And even if some legislation tries to put a stop to it, companion animals are often kept in isolation from conspecifics, even highly social animals, like parrots, which has been shown to have harming effects (Aydinonat et al. 2014). Furthermore, companion animals kept in shelters, such as dogs, might very often experience the breaking up of social relationships when individuals of their group are rehomed. In this light, the common practice of rescuing dogs from the streets may not be as innocuous as is usually considered, as these animals lose their familiar environment and very likely all their well-known partners. They might be brought to a foster home with no other companion dog around them—a situation that could possibly mean fewer opportunities for their moral capabilities to be exercised.

In sum, if the animals in these different examples are subjects with complex social lives that include moral lives, then re-grouping, separating, and isolating them may disrupt or preclude the appearance of those bonds that are a pre-condition for the exercise of their moral capabilities.

### Practices That Eliminate the Moral Capabilities of Animals

In the practices that we have considered until now, the animals involved are, to a bigger or lesser extent, prevented from exercising their moral capabilities. In this final section, we will consider human practices that go over and beyond this, by altogether eliminating the animals’ capability to behave morally.

Some human–animal interactions involve breeding, training, conditioning, or modifying the animals with the aim of eliminating some (or all) of their moral capabilities. The most obvious example here is that of fighting animals. Indeed, the training (and also breeding^21^) that fighting dogs undergo aims precisely at enhancing their aggressiveness and eliminating any potential caring response to a conspecific in distress (Kalof and Iliopoulou 2011). Cattle used for bullfighting are also selectively bred to enhance aggressiveness (Silva et al. 2006; Correia et al. 2015), as are the chickens used for cock fighting (Guo et al. 2016).

But it is probably in the lab where the elimination of animals’ moral capabilities has been performed in the most intentional and methodical manner. Indeed, several psychologists have undertaken this as a research project. Perhaps the most paradigmatic example is Harry Harlow and his experiments on maternal separation, dependency needs, and social deprivation (for an overview on Harlow’s research see Harlow 1958; Harlow et al. 1965; Blum 2004). Harlow raised rhesus monkeys from birth onward in bare wire cages, facing partial or total maternal deprivation. He would offer them the choice between two inanimate surrogate mothers: one made of cloth and the other of wire. The infants were found to insistently seek the cloth mothers, even when they were designed to shake them, stab them with blunt spikes, or push them away via a mechanical flap. These monkeys “never experienced mother love, nor any other kind of monkey affection,” and when they themselves were impregnated and had their own offspring, they “either completely ignored or abused” their babies, or in many cases killed them (Harlow and Suomi 1971, 1535). By subjecting monkeys to this treatment, as well as to total isolation chambers, Harlow systematically created individuals who were completely “deficient in social play and sexual behavior,” as well as “hyperaggressive in peer interaction” (Arling and Harlow 1967, 371).

Harlow’s experiments, as shocking as they sound, should not be considered an isolated event in psychology. During the past 30 years, on-going maternal deprivation experiments, conducted for example at the NIH in the US, have subjected hundreds of infant macaques to similar conditions as Harlow’s experiments. They are heavily criticised from a bioethical perspective for being both unnecessary and cruel (Novak 2014; Medical Research Modernization Committee 2017). The potential elimination of the monkeys’ moral capabilities is another factor to consider in the ethical assessment of these tests.

Further examples of experiments performed with the aim of interfering in the animals’ moral capabilities include Tulogdi et al. (2014), who subjected rats to post-weaning social isolation, thereby inducing deficits in pro-social behaviour. These deficits were eliminated by resocialisation during adulthood, but the rats’ abnormal aggressiveness remained resilient to this treatment. Hernandez-Lallement et al. (2016) have also induced deficits in pro-social behaviour in rats by performing surgery aimed at damaging the amygdala (brain area responsible for emotion and affiliation). They plan to continue their research along these lines in order to produce “an animal model of callousness,” for which “[r]odents offer a cheap, convenient and ethically less controversial alternative to non-human primates” (Hernandez-Lallement et al. 2018). Our argument helps to shed some light on the ethical problems that are, in fact, involved in such experiments.

As in the hypothetical example of Sustitia_3_, some human practices may destroy the moral capabilities of animals indirectly, as an unintended side effect of a treatment that has other aims. For example, there have been reports of rats becoming habituated to distress cues from conspecifics after repeated exposure to them during pro-sociality experiments (Church 1959). The mother-infant relationship is also artificially terminated in the case of many farm, zoo, and lab animals, as well as for some companion animals, e.g., if puppies are sold before they reach an appropriate age. As the Harlow experiments exemplify, the absence of an appropriate mother-infant bond may have profound effects on the development of the moral capabilities of animals. And lastly, the social isolation that can be found in many farms, zoos, labs, and even some households, may not just prevent the animals from exercising their moral capabilities, but also, in the long run, effectively eliminate their capacity to care for others. The humans who are causally responsible should, arguably, be blamed for this harm, even if it was unintended. This would especially be the case if the humans were aware of this side effect, and simply assumed it as an inevitable consequence.

## Conclusion

When we initially set out to investigate the ethical implications of considering that some animals are moral subjects, we expected it to be a conceptual exploration, with little relevance outside the proverbial armchair. Rather the opposite turned out to be the case. We have found many contexts, including routine procedures in farms, labs, and in our homes, where humans potentially interfere with, hinder, or destroy the moral capabilities of animals. And by opening up to other normative theories besides the capabilities approach we could perhaps find further examples. We leave that to future research, and hope to at least have given a good sense of how the possibility of moral subjecthood in animals creates conceptual space for a type of harm that has been little, if at all, discussed, and that may be very real and important. Whether or not welfarists can find a way of capturing this harm remains to be seen, but we hope to have convincingly shown how, in its purely hedonistic formulation, it is unlikely that welfarism can account for this harm.

## Footnotes

1 The idea that some animals have some degree of moral agency has also been defended by other authors, such as Sapontzis (1987), Pluhar (1995), De Grazia (1996), Shapiro (2006) and Andrews and Gruen (2014). We focus on Rowlands’ approach for present purposes because it is the most detailed philosophical account of animal morality to date, and also because we find the distinction between moral subjects and moral agents to be very useful when discussing animal morality.

2 In addition to these systematic studies, which document animals engaging in apparently moral behaviour towards both humans and conspecifics, one can easily come across many relevant anecdotes. For instance, there are reports of dolphins helping other dolphins (Park et al. 2012), and even humans (Bekoff and Pierce 2009, 108).

3 While the possibility of animal morality is gaining increasing support from scholars, there are authors who have expressed dissenting views, on both empirical and conceptual grounds. There are, for instance, empirical critiques of the ‘fairness’ experiments by Brosnan and De Waal (e.g. Penn et al. 2008), as well as the ‘empathy’ studies on rodents (e.g. Schwartz et al. 2017). There are also conceptual critiques that focus on whether these studies provide evidence of specifically moral motivations (see e.g. Korsgaard 2006; Carron 2018 for critiques of De Waal’s interpretation of the relevant studies). For a comprehensive overview of the conceptual disagreements in the animal morality debate, see Fitzpatrick (2017). For an up-to-date discussion of the empirical evidence and the debates on how to interpret it, see Andrews and Monsó (in preparation).

4 This line of thinking is deeply rooted in different cultures. In the Judeo-Christian tradition, for example, human superiority has traditionally been interpreted as granting a right to dominate and exploit nature. Some modern theologians, however, argue that human superiority should instead be interpreted as implying a duty of stewardship (e.g. Linzey and Cohn-Sherbok 1997).

5 One could object here by saying that full moral status depends on full-fledged moral agency, which can be plausibly regarded as an exclusively human domain (barring any extraterrestrial moral agents). However, we assume that anyone who defends this position would be unable to satisfactorily address the so-called ‘marginal cases’ objection, namely, that some humans don’t possess full-fledged moral agency, due to age or impairment, and yet we still want to grant them full moral status.

6 We use the term ‘well-being’ to refer, broadly, to how well life is going for an individual. We thus take welfarism to be a monistic theory that equates well-being with positive hedonistic welfare, and we understand pluralistic theories as defending the existence of other prudential goods that contribute to well-being.

7 Following Schmidt (2011, 158), welfare scientists can be broadly categorised into those that focus on the *subjective* aspects of animal well-being (e.g. Duncan 1993) and those that focus on *objective* aspects, such as biological functioning, the ability to cope with the environment (e.g. Broom 1991), or quantifiable measures of animal welfare such as behavioural indicators (e.g. Dawkins 2006). However, there are also welfare scientists that stress both (e.g. Webster 2005). Our critique of welfarism in this paper is directed at those scholars that focus on the subjective aspects. It is important to note, though, that in animal welfare science subjective quality of life is often considered a very dominant component of animal well-being.

8 Even though Sustitia_1_ is a hypothetical example, her life conditions do not differ much from those of sows in industrial farms. See EFSA (2007) for a comprehensive review of the welfare problems involved in pig husbandry.

9 What constitutes *unnecessary* suffering is, for sure, debatable. Farm animals are routinely subjected to a series of painful or stressful procedures that are deemed necessary out of economic interests. Farrowing crates, for instance, are used to prevent the sows from rolling over and crushing the piglets. However, studies have shown that sows kept in an enriched environment, with more space and access to straw and sand, are less likely to crush their piglets and more likely to respond to a piglet’s distress cries by standing up (Herskin et al. 1998). Providing such environmental enrichment, together with the extra space required, would entail extra costs for the farmers, thus ultimately raising the price of pork. Are farrowing crates then “necessary”? It depends on the normative ethics one subscribes to. From the perspective of some utilitarian welfare ethics, it might be permissible to weigh the sows’ interest in not suffering against the farmers’ interest in earning a living. In contrast, from the perspective of other ethical accounts (including other consequentialist approaches, and for sure an animal rights approach), this might not be permissible, as the interest in not suffering ranks in principle higher than economic interests (at least as long as the latter do not constitute a matter of life or death for the farmer or consumer).

10 In this respect, these hypothetical piglets are not so different from real piglets, as these are all routine procedures in livestock management (RSPCA 2016).

11 Sustitia_2_ may share this capacity with actual pigs. Recent studies suggest that pigs possess a capacity for emotional contagion, for they tend to display behaviours associated with negative emotions (e.g. escape attempts, defecation) or positive ones (e.g. play behaviour, barks) depending on whether they are paired with a conspecific who has undergone a negative or a positive treatment, respectively (Reimert et al. 2013; Reimert et al. 2015; Goumon and Špinka 2016). In fact, she also appears to share this capacity with chimpanzees (Parr 2001), geese (Wascher et al. 2008), dogs (Yong and Ruffman 2014; Huber et al. 2017), mice (Langford et al. 2006; Jeon et al. 2010), rats (Knapska et al. 2006; Atsak et al. 2011), prairie voles (Burkett et al. 2016), and chickens (Edgar et al. 2011). Studies on emotional contagion often involve animals undergoing negative stimuli in order to determine whether the witnessing animals get stressed, too. While this is an issue that goes beyond the scope of this paper, we would like to note that these sorts of experiments may be ethically problematic and should not remain unquestioned.

12 When referring to Sustitia_2_’s mechanism as her ‘sympathy,’ we are following a trend within the animal morality debate that considers sympathy to be a form of empathy that entails the (partial) adoption of another’s emotional state, together with a possession of a clear self-other distinction, an understanding that the other is in need or suffering, and an intention to ameliorate the other’s situation (see, e.g., de Waal 2008, 283). There is, however, a big definitional debate surrounding both sympathy and empathy, which we do not intend to take a stand on. Referring to Sustitia_2_’s mechanism as ‘sympathy’ is meant for ease of exposition, but we do not necessarily subscribe to any particular definition of this term.

13 For a detailed explanation of the importance of these traits from a moral perspective, see Rowlands (2012); also Monsó (2017).

14 Indeed, this is the usual effect when animals engage in affiliative behaviour directed at a distressed conspecific (see, e.g., Kikusui et al. 2001; Fraser et al. 2008; Clay and de Waal 2013; Smith and Wang 2014; Burkett et al. 2016).

15 In fact, the authors themselves hint at this idea (Purves and Delon 2018, 329).

16 Note that this is a conceptual, not empirical, claim. In the ‘real world,’ the thwarting of a being’s basic capabilities may always lead to pain or suffering, but there is no necessary connection between the two, as we shall discuss in the section Theoretical Implications: Moving Beyond Welfarism.

17 One could object that this first consideration is question-begging. Of course, in a sense, it is, but that does not necessarily render it useless. By putting forward our intuition that Sustitia_2_ is harmed in two different ways, we are making explicit a claim that a welfarist would have to renounce. Those readers who share our intuition may find this a compelling reason to set welfarism aside.

18 This is not an unrealistic trait of Sustitia_2_’s. Rats given anxiolytics have been found less likely to help a conspecific in need (Ben-Ami Bartal et al. 2016), so distress may be a crucial component of some of the moral motivations of animals.

19 We deliberately point to this because a possible counter-argument might be to say that this implies that the ethical problem of piglet castration could be solved if the sows were allowed to comfort them afterwards. Of course, we believe that such a counter-argument is flawed. There are several further considerations that need to be taken into account. For one, the piglets’ suffering would remain and would be an ethical problem even if the sows were allowed to comfort them. In addition, witnessing the suffering of piglets is probably not an isolated event for most sows but something they have to face rather often. A sow currently gives birth to 10–16 piglets per litter, producing 25–30 pigs per year (Kim et al. 2013). All male piglets are normally castrated without pain relief. Additionally, most piglets undergo other painful routine procedures like teeth-clipping or tail-docking, abrupt weaning, re-grouping and transport. All of these procedures are stressful, the witnessing animals cannot escape them, and “the chance of being affected by the distress of their group members is therefore relatively high” (Reimert et al. 2015, 519). The welfare problem might be so big as to outweigh any considerations regarding the intrinsic value of caring for others. But even if we were to consider the sows’ caring behaviour as intrinsically valuable in any case, a proper ethical assessment of the situation would take into account the circumstances that originate this behaviour, which are ethically dubious in many respects.

20 See Thompson (2008) and Ferrari (2012) for an overview of this debate.

21 The case of breeding is peculiar, insofar as selectively breeding for a certain characteristic cannot, in virtue of the non-identity problem, be considered an instance of changing an *individual*’s capabilities. Instead, we would have to say that selective breeding changes the capabilities of the *species* or the *population*. We believe that this is nevertheless an instance of harm, because of the intentional manner in which this breeding is performed and the goal behind it. We are assuming that the capabilities approach can accommodate harm inflicted on the capabilities of populations, as well as individuals, but this is a matter that merits further discussion.
